# Potentially Toxic Elements in Urban Soils from Public-Access Areas in the Rapidly Growing Megacity of Lagos, Nigeria

**DOI:** 10.3390/toxics10040154

**Published:** 2022-03-23

**Authors:** Abimbola O. Famuyiwa, Christine M. Davidson, Sesugh Ande, Aderonke O. Oyeyiola

**Affiliations:** 1 Department of Pure and Applied Chemistry, University of Strathclyde, 295 Cathedral Street, Glasgow G1 1XL, UK; abimbola.famuyiwa@gmail.com (A.O.F.); sesughande@gmail.com (S.A.); 2 Department of Science Laboratory Technology, Moshood Abiola Polytechnic, Abeokuta, Ogun State, Nigeria; 3 Department of Chemistry, University of Agriculture, Makurdi, Benue State, Nigeria; 4 Department of Chemistry, University of Lagos, Akoka-Yaba, Lagos State, Nigeria; aderonkeoyeyiola@gmail.com

**Keywords:** soil contamination, heavy metals, sequential extraction, health risk assessment

## Abstract

Rapid urbanization can lead to significant environmental contamination with potentially toxic elements (PTEs). This is of concern because PTEs are accumulative, persistent, and can have detrimental effects on human health. Urban soil samples were obtained from parks, ornamental gardens, roadsides, railway terminals and locations close to industrial estates and dumpsites within the Lagos metropolis. Chromium, Cu, Fe, Mn, Ni, Pb and Zn concentrations were determined using inductively coupled plasma mass spectrometry following sample digestion with aqua regia and application of the BCR sequential extraction procedure. A wide range of analyte concentrations was found—Cr, 19–1830 mg/kg; Cu, 8–11,700 mg/kg; Fe, 7460–166,000 mg/kg; Mn, 135–6100 mg/kg; Ni, 4–1050 mg/kg; Pb, 10–4340 mg/kg; and Zn, 61–5620 mg/kg—with high levels in areas close to industrial plants and dumpsites. The proportions of analytes released in the first three steps of the sequential extraction were Fe (16%) < Cr (30%) < Ni (46%) < Mn (63%) < Cu (78%) < Zn (80%) < Pb (84%), indicating that there is considerable scope for PTE (re)mobilization. Human health risk assessment indicated non-carcinogenic risk for children and carcinogenic risk for both children and adults. Further monitoring of PTE in the Lagos urban environment is therefore recommended.

## 1. Introduction

Urban soil pollution has become a major environmental concern in recent decades. Increased migration from rural to urban areas, in particular in the developing world, has resulted in high population density and rapid increase in anthropogenic activities [1]. Over half of the world’s population now lives in urban areas [2], and this puts substantial pressure on environmental resources, such as soil and water. 

With a population of at least 20 million people and an estimated population growth rate of about 600,000 persons per annum, Lagos is one of the most densely populated cities in the world [3]. Within the metropolis, there is rapid industrialization, continuous infrastructural development, and a high prevalence of vehicular traffic congestion. Incessant demand for land means that recreational open spaces can be found in proximity to dumpsites, and schools and housing are often co-located with industrial estates. Therefore, Lagos residents are subjected to an array of potential pollution sources that may have adverse effects on their health.

Numerous studies have reported evidence of anthropogenic inputs of potentially toxic elements (PTE) to urban soils. Extensive work has been carried out in developed countries [4,5,6,7]. Less attention has been paid to urban soils of developing nations, although their importance is increasingly being recognized [8,9,10,11,12]. Potentially toxic elements are one of the most studied soil contaminants because they are ubiquitous and persistent. Metals are non-biodegradable and accumulative in nature; emission and deposition over a long period of time can lead to enrichment in surface environments. The prolonged presence of PTEs in urban soils, together with their proximity to human populations, can lead to exposure via inhalation, ingestion, and dermal contact [13,14]. Because these contaminants become hazardous when present in soil above certain concentrations, this can have significant health implications.

Measurement of total or pseudo-total (aqua-regia soluble) PTE concentrations in soil can provide important information about distribution and enrichment, but it is likely to overestimate human health risk because only a fraction of the total content is usually bioavailable [15]. The mobility and availability of PTEs in soil depends on complex interactions between multiple factors, including solubility, the availability of binding sites, complexation, pH and redox processes [16]. Sequential extraction involves the treatment of solid environmental samples with a series of reagents to partition PTE content into various fractions, nominally corresponding to major soil mineral phases but more accurately representing operationally defined reservoirs with the potential to become mobile under changes in environmental conditions, such as pH and redox potential. Several sequential extraction schemes have been developed and applied [17,18,19,20]. Amongst the most popular is the harmonized Community Bureau of Reference of the European Commission (BCR) sequential extraction procedure [21], summarised in Table 1. Advantages of this protocol are that it incorporates an internal quality check—comparison of the sum of the steps, Σ(step 1–4), with results of a separate pseudo-total digestion—and that dedicated certified reference material (CRM)—BCR 701—is available.

Previous studies have investigated PTE in urban soils of Lagos State. However, their scope has either been limited to a particular land use—for example, roadside soils [22,23] or soils from school playgrounds [8,24]—or featured assessment of contamination in proximity to specific industrial plants [25], dumpsites [26] or electronic waste (e-waste) processing sites [27,28,29]. Much of the work is at least a decade old [22,23,24,25] and therefore may not accurately reflect the current status of the rapidly growing metropolis. Few works have considered PTE mobility. Adeyi et al. [30] applied Tessier sequential extraction [17] to residential soils from Lagos and Ibadan as part of their study of the potential health impacts of Cd and Pb associated with the use of metal-rich paints. Oyeyiola et al. [31] employed the BCR procedure [21] in their investigation of partitioning, mobility and ecotoxicology of Cd, Cr, Cu, Pb and Zn in sediment from the Lagos Lagoon and three trans-urban rivers. However, there is a need for more comprehensive evaluation of both levels and potential mobility of PTE in Lagos urban soils, in particular soils that local citizens are most likely to interact with.

The aims of the current study were therefore to determine the concentrations and potential mobilities of Cr, Cu, Fe, Mn, Ni, Pb and Zn in soils from public-access areas across the Lagos metropolis and to evaluate the risks associated with human exposure to PTE in Lagos urban soils.

## 2. Materials and Methods

### 2.1. Study Area

Lagos lies in the Nigerian sector of the Dahomey (or Benin) basin. The area is characterized by sediments of Cretaceous to recent origin underlain by Precambrian basement rocks of granitic composition [32]. The Cretaceous and Tertiary sediments include sands, marine shales and limestone. Quaternary sediments consist of coastal plain sands (>100 m in thickness) with alluvial deposits in the river valleys. Lagos is Nigeria’s most populous city and the seventh fastest-growing city in the world. Lagos State Government estimated the population of Lagos as 17.5 million during a parallel census conducted in 2006, with more than 12 million people living in the urban areas. A more recent report [3] estimated its population as 21 million, making Lagos the largest city in Africa. Lagos experiences rainy and dry seasons, with the latter accompanied by hot, dry and dusty winds. It represents the most industrialized area in Nigeria, with over 60% of total industrial activities.

### 2.2. Sampling and Sample Preparation

**Figure 1:**
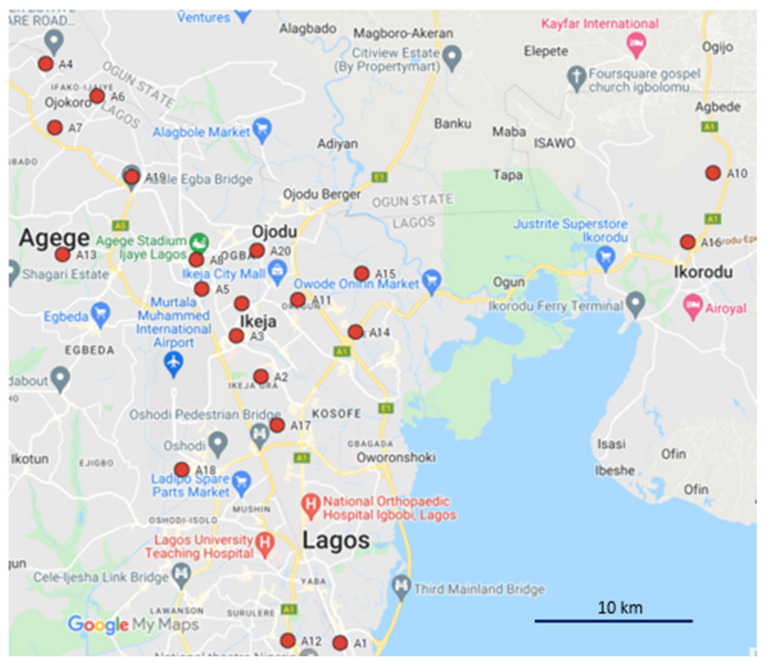
Map of the Lagos area showing sampling points (prepared using Google Maps).

We selected 20 urban from a larger set of 92 samples collected as part of a previous study [33]. Sampling locations are shown in Figure 1. These included examples of different types of public-access areas (parks and open spaces, ornamental gardens, roadsides, industrial estates, railway terminals and locations in the vicinity of dumpsites). At each sampling location, a composite soil sample was collected to a depth of 10 cm. This consisted of 4–8 sub-samples taken 2 m apart in a square grid (the number of sub-samples depending on the shape of the area). Grass, leaves, paper and plastic debris present in the samples were discarded. Wet soil samples were air-dried for 3 days in the laboratory of the Lagos Ministry of Environment. Then, approximately 500 g of each soil was placed in a sealed, labelled polythene bag and transported from Lagos, Nigeria to the University of Strathclyde, Scotland, UK under a Scottish Government soil import license (IMP/SOIL/24/2014) for further processing and analysis.

On arrival, the soil samples were air-dried for 14 days, and then sieved through a 2 mm nylon mesh sieve before grinding and homogenization with mortar and pestle. Test portions for digestion or extraction were obtained by coning and quartering. All glass and plasticware was soaked in 5% (*v*/*v*) nitric acid overnight (general-purpose-grade reagent, Sigma Aldrich, Gillingham, UK) and then washed thoroughly with distilled water before use. 

### 2.3. Microwave-Assisted Pseudo-total Digestion

Samples were digested with aqua regia prepared by mixing extra-pure hydrochloric (HCl) and nitric (HNO_3_) acids (Sigma-Aldrich, Gillingham, UK) in the ratio 3:1 (*v*/*v*). Each soil sample (1 g) was weighed into a high-pressure vessel, and 20 mL of freshly prepared aqua regia was added. This was left to stand overnight in a fume cupboard to allow any vigorous reaction to subside. Then, the vessels were placed in a MarsXpress^TM^ (CEM Microwave Technology, Ltd., Middle Slade, Buckingham, UK) microwave digestion system and heated to 160 °C using 1600 W power for 40 min (comprising 20 min ramp to temperature and 20 min hold at temperature). Digests were then allowed to cool and were filtered. Filtrates were made up to 100 mL with deionised water and stored at 4 °C in a refrigerator prior to analysis. Replicate samples (*n* = 3) were digested, along with procedural blanks.

### 2.4. Sequential Extraction

The BCR sequential extraction procedure was carried out as described by Rauret et al. [21]. The experimental protocol is summarised below. Samples were analysed in triplicate, along with procedural blanks.

Step 1: Exchangeable phase

Approximately 1 g of soil was weighed into a 100 mL centrifuge tube, and 40 mL of 0.11 M acetic acid added. The mixture was shaken for 16 h (overnight) using a GFL 3040 mechanical end-over-end shaker (GFL, Burgwedel, Germany). The extract was separated from the residue by centrifuging at 3000× *g* for 20 min in an Allegra 21 centrifuge (Beckman Coulter Ltd., High Wycombe, UK). The supernatant was decanted and stored in a polyethylene bottle at 4 °C in a refrigerator prior to analysis. The residue was washed by adding 20 mL of distilled water and shaking for 15 min. Following centrifugation, the supernatant was decanted and discarded.

Step 2: Reducible phase

A volume of 40 mL of freshly prepared 0.5 M hydroxylamine hydrochloride solution was added to the washed residue from step 1 in the same centrifuge tube. The mixture was shaken and centrifuged, the supernatant was recovered, and the residue was washed, as described in step 1.

Step 3: Oxidisable phase

A volume of 10 mL of 8.8 M hydrogen peroxide solution was added slowly, in small aliquots to avoid losses due to possible violent reaction, to the washed residue from step 2. The centrifuge tube was loosely covered with its cap, and the contents were digested at room temperature for 1 h with occasional manual shaking. The digestion was continued for another 1 h at 85 ± 2 °C in a water bath, with occasional manual shaking for the first 30 min. Then, the sample mixture was reduced in volume to about 3 mL by further heating the uncovered tube. Another 10 mL of hydrogen peroxide solution (8.8 M) was added, and the covered sample was heated for a further 1 h at 85 ± 2 °C. Subsequently, the cap of the centrifuge tube was removed, and the volume was reduced to about 1 mL, with care not to take to complete dryness. A volume of 50 mL of 1.0 M ammonium acetate solution was added to the cool, moist residue, and the mixture was shaken for 16 h (overnight). The sample was centrifuged, and the supernatant was recovered as described in step 1.

Step 4: Residual phase

A volume of 20 mL of aqua regia was used to wash the residue from step 3 into a pressure vessel, where it was digested as described in Section 2.3.

### 2.5. Analysis of Sample Digests and Extracts

Analyte concentrations were measured in soil digests and extracts using a Model 7700x inductively coupled plasma mass spectrometry system (Agilent Technologies, Cheadle, UK). Commercially available stock solutions (from Qmx Laboratories, Thaxted, UK) were used to prepare reagent-matched multielement standard solutions for instrument calibration in the range of 0–1600 µg/L for Cr, Cu, Mn, Pb, Ni and Zn and 0–100,000 µg/L for Fe. The internal standard was ^115^In. Before each batch of analyses, the instrument was tuned to verify mass resolution and maximise sensitivity. Collision cell technology mode was used for the determination of ^52^Cr, ^63^Cu, ^56^Fe, ^55^Mn, ^60^Ni, ^208^Pb and ^64^Zn. During analysis, a mid-range calibration standard (800 µg/L) was checked after every tenth sample measured. The calibration curves for the determined PTEs gave a linear fit with regression coefficient of at least 0.999.

### 2.6. Determination of Soil pH and Organic Matter

pH was determined in a suspension of 5 g soil in 25 mL of deionised water using a pH meter (SG2-ELK-SevenGO^TM^ pH, Mettler Toledo, Leicester, UK) according to the British standard method [34]. Soil organic matter was estimated by loss on ignition of dry matter [35]. A muffle furnace (Elite Thermal Systems Box Furnace, model number BSF12/6-2416CG, Market Harborough, UK) ramped at 10 °C per min and held at 550 °C for 8 h was used for this purpose.

### 2.7. Quality Control

Analytical quality was assessed using CRMs BCR 143R (sewage sludge amended soil) for pseudo-total digestion and BCR 701 (lake sediment) for sequential extraction (Table 2).

Agreement between found and certified values for BCR 143R was excellent (100 ± 5%). For BCR 701, the recoveries of PTEs in exchangeable and reducible phases were 100 ± 15%, whereas recoveries in the oxidisable and residual phases were generally 100 ± 30%, except for Pb in step 3 (32%). In his review of results reported for BCR 701 over a 10-year period, Sutherland [36] highlighted other instances of low Pb recovery (<50% of the certified value) in step 3, together with the high degree of imprecision associated with the measurement of Pb in this step during the BCR 701 certification process. Overall, recoveries tended to be low in step 3, relative to certified or indicative values, but high in step 4. However, summations of the amounts of analyte released in steps 1 to 4 of the sequential extraction agreed (within ±20%) with results of pseudo-total measurement in BCR 701. This suggests that the quality of the extraction was adequate.

### 2.8. Potential Health Risk of PTE

Potential non-carcinogenic and carcinogenic health risks were determined using United States Environmental Protection Agency [37,38,39,40] methods and exposure parameters recommended for management of contaminated land in South Africa [41]. Exposure assessment was carried out by calculating the average daily intake (*ADI*) of each PTE through ingestion, inhalation and dermal contact for adults and children (Equations (1)–(3)). Adults and children are considered separately because of their behavioural and physiological differences [42].

(1) $$ADI_{ing}=\frac{C\times IR\times EF\times ED}{BW\times AT}\times 10^{-6\;}$$

(2) $$ADI_{inh}=\frac{C\times Inh\times EF\times ED}{PEF\times BW\times AT}\;$$

(3) $$ADI_{dermal}=\frac{C\times SA\times AF\times ABS\times EF\times ED}{BW\times AT}\times 10^{-6}$$

where *ADI_ing_* is the average daily intake of a PTE from soil via ingestion in mg per kg per day (mg/kg/day), *C* is the concentration of PTE in the soil in mg/kg, *IR* is the ingestion rate, *EF* is the exposure frequency, *ED* is the exposure duration, *BW* is the body weight of the exposed individual and *AT* is the time period over which the dose is averaged. *ADI_inh_* is the average daily intake of PTE from soil via inhalation in mg/kg/day, *Inh* is the inhalation rate and *PEF* is the particulate emission factor. *ADI_dermal_* is the average daily intake of PTE from soil via dermal contact in mg/kg/day, *SA* is the skin surface area, *AF* is the soil-to-skin adherence factor and *ABS* is the fraction of the applied dose absorbed across the skin.

Hazard quotient (HQ) and hazard index (HI) were used to estimate the non-carcinogenic risk of PTEs in soil [40]. HQ characterizes the health risk of non-carcinogenic adverse effects due to exposure to toxicants and is defined as the quotient of ADI or dose divided by the toxicity threshold value, which is referred to as the chronic reference dose (RfD) in mg/kg/day for a specific PTE, as shown in Equation (4).

HQ = ADI/RfD(4)

To assess the overall potential of non-carcinogenic effects posed by a PTE, the calculated values of HQ are summed to give HI (Equation (5)).

HI = HQ_ing_ + HQ_inh_ + HQ_dermal_ (5)

For carcinogens, the risks are estimated as the incremental probability of an individual developing cancer over a lifetime (assumed to be 70 years) because of exposure to the potential carcinogen, CR, calculated using Equation (6).

CR = ADI × CSF(6)

where CSF (mg/kg/day) is the cancer slope factor, which converts the estimated daily intake of a PTE to an incremental risk of an individual developing cancer [39]. The total excess lifetime cancer risk for an individual is ultimately calculated from the average contribution of the individual PTE across all exposure pathways using Equation (7).

CR_total_ = CR_ing_ + CR_inh_ + CR_dermal_ (7)

where CR_ing_, CR_inh_, and CR_dermal_ are risk contributions through ingestion, inhalation, and dermal pathways, respectively.

Values for all of the parameters used in the risk calculations are presented in Table 3.

## 3. Results and Discussion

### 3.1. Soil Characterstics and Pseudo-total PTE Concentrations

The pH of the soil samples ranged from 5.8 to 10, with an average of 7.5 (Table 4). Loss on ignition (LOI) values were low (0.07–5.0, with an average of 1.5). This is consistent with the hot and humid climatic conditions in Lagos, which typically deplete soil organic matter [43]. Findings were in agreement with previous work on Lagos residential soils [30] and playgrounds [24], which generally reported organic matter content <2% and slightly alkaline pH values.

Pseudo-total PTE concentrations (Table 4) reflected varying degrees of soil contamination, as expected, given the various types of land use represented. Samples A1 to A7, obtained from gardens and open spaces, were less contaminated than samples A8 to A20, (soil from industrial estates; A8 to A10), railway terminals (A11 to A13) and dumpsites (A14 to A20). Soil guideline values have not yet been defined specifically for use in Nigeria. However, compared with the frequently cited Dutch soil quality standards [44], more than half of the samples contained Cu, Pb and Zn (the ’urban metals’) [45,46] at concentrations greater than target values [44], whereas three soils—A10, A16 and A19—contained all three elements at concentrations sometimes considerably in excess of the intervention values [44]. The first of these, soil A10, was from an industrial estate where high PTE levels may be attributed to emissions from zinc smelting, steel production and metal foundry plants. Soils A16 and A19 were from the vicinity of dumpsites. Typical dumpsites in Lagos receive large volumes of domestic, industrial and e-waste daily, and there are also a number of auto repair workshops in proximity to these specific locations, all of which are likely to have contributed to the enhanced soil PTE contents observed. 

As mentioned above, literature data on Lagos soils is limited to values for a few elements measured at a few sites. However, average concentrations found in the current study were higher than those reported in previous studies [22,23,24,25,26,30], which likely represents PTE accumulation over time as urbanization and industrialization have progressed. Exceptions were some high Cu, Pb and Zn concentrations reported recently at e-waste recycling locations in Owutu, Ikorodu [28] and Alaba International Market [29], which were of a similar magnitude to results for sites A16 and A19. Levels of PTE in the current study were generally substantially higher than PTE measured in urban soils of cities in other developing countries, such as Kampala, Uganda [47]; Karachi, Pakistan [48]; and Sunyani, Ghana [49].

### 3.2. Sequential Extraction and PTE Mobility

Sequential extraction was performed on the soil samples to assess the potential for PTE (re)mobilization. Results are presented in Supplementary Tables S1–S7. To check the quality of the data obtained, the amounts of analyte recovered—Σ(step 1–4)—were compared with those released by aqua regia digestion. A total of 57 (of 140) of the sequential extraction results fell within 10% of corresponding pseudo-total values. A further 63 were either 70–90% or 110–130% of pseudo-total concentrations, i.e., overall, in 86% of cases, the sum of the steps of the sequential extractions was 100 ± 30% of the aqua regia-soluble content. Only three recoveries were either <50% or >150% of the aqua regia-soluble content: Ni at site A20 (39%) and Pb at sites A1 (48%) and A11 (169%). Site A20 is close to a foundry, and it is possible that there are metal-rich particles heterogeneously distributed within it. This may also be the case for Pb at A11. As well as a railway, this site is close to a major bus depot where mechanical work is undertaken (including removal and servicing of vehicle batteries). Site A1 is an open space in a wealthier part of Lagos metropolis and not highly contaminated with PTEs. There is a relatively large uncertainty in the Pb pseudo-total concentration (17%, *n* = 3) and hence in the recovery calculated. 

**Figure 2:**
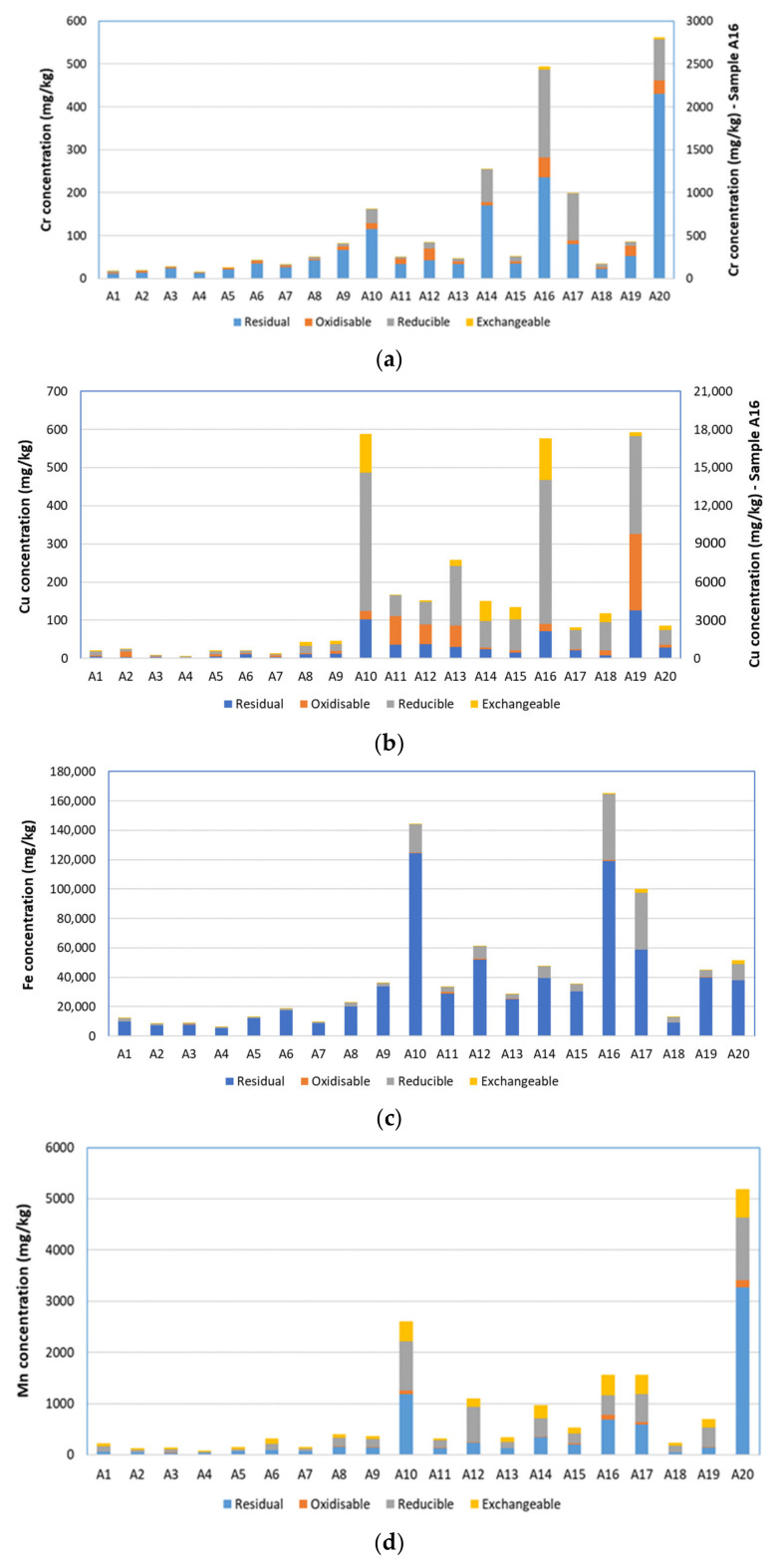
Fractionation of (**a**) Cr, (**b**) Cu, (**c**) Fe and (**d**) Mn according to BCR sequential extraction. Note that Cr and Cu are plotted against a secondary axis for site A16.

**Figure 3:**
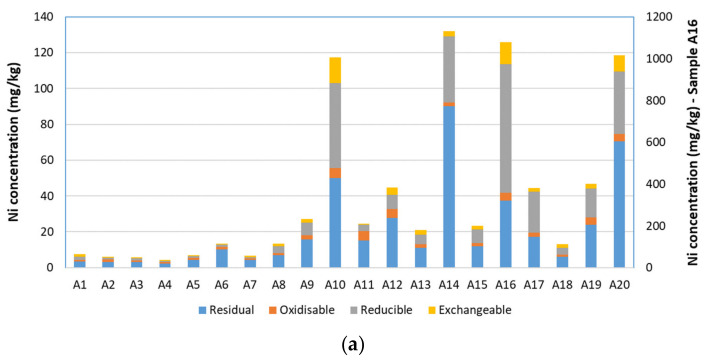

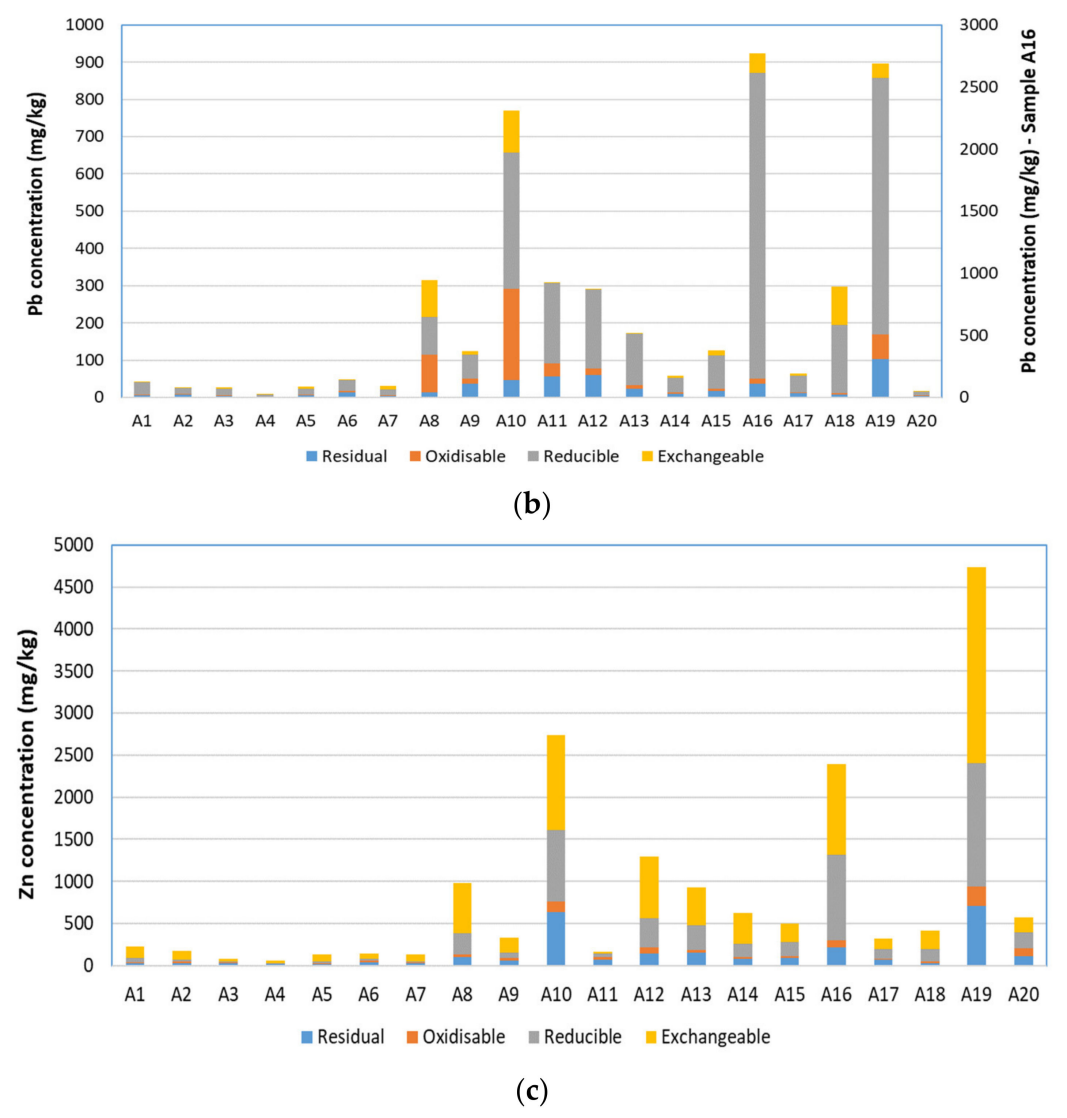
Fractionation of (**a**) Ni, (**b**) Pb and (**c**) Zn according to BCR sequential extraction. Note that Ni and Pb are plotted against a secondary axis for site A16.

The fractionation patterns obtained using the BCR procedure are shown in Figure 2 and Figure 3. Chromium was predominantly associated with step 4, the residual phase, in most of the samples (Figure 2a). Three-quarters of the soils studied contained more than 70% of their Cr content in the residual phase. This is in agreement with other urban soil studies [50,51,52]. Wu et al. [53] found 92% of Cr in the residual phase of urban soils of Guiyang City, China. The presence of Cr in residual forms suggests that the element is strongly bound to soil minerals; therefore, mobilization is unlikely to occur under typical environmental conditions. However, where Cr concentrations were highest, a larger proportion was associated with the reducible phase (step 2). This is worrisome because it indicates that were the soil to become waterlogged and anoxic, there is potential for Cr remobilization due to reduction and dissolution of iron and manganese oxyhydroxides.

Copper was released at various steps in the sequential extraction (Figure 2b), with the highest proportion associated with the reducible phase in most samples. The association of Cu with iron and manganese oxides and hydroxides—the target phase of step 2 of the BCR protocol—has been well documented in polluted urban soils, dusts and sediments [50,54,55,56,57,58,59]. For some samples, an appreciable amount of Cu was also released in step 2 which, again, has been reported in previous studies; for example, Szolnoki et al. [55] found 24% of Cu in the oxidisable fraction of urban vegetable garden soils from Szeged, Hungary. Because the majority of the overall Cu content was in non-residual forms, there is considerable potential for Cu mobilization under changing environmental conditions. Of particular concern is the most contaminated dumpsite, A16, where >3000 mg/kg of Cu was found in the easily mobilized exchangeable phase.

Almost all the Fe in Lagos urban soils was associated with the reducible and residual phases (Figure 2c), which was expected because step 2 of the BCR protocol targets iron and manganese oxyhydroxides, and ferrous minerals constitute a major structural component of soil. Similar findings have been reported for urban soils from public-access areas of five European cities [52], as well as urban vegetable garden soil [60]. The predominance of Fe in the residual phase indicates low mobility and bioavailability.

Manganese was found in all four phases (Figure 2d), with residual and reducible forms generally dominating for similar reasons to Fe. Manganese is one of the most abundant elements in the earth crust [57], and the hydroxylamine–hydrochloride reagent employed in step 2 principally targets Fe-Mn oxyhydroxides. Previous work on urban soils from five European cities reported [52] a similar manganese distribution between the fractions defined by sequential extraction. Significant amounts of Mn were also located in the most labile, exchangeable phase. This suggests that Mn is relatively mobile in Lagos soils.

Nickel was mainly associated with the reducible and residual phases (Figure 3a). This is in agreement with sequential extraction results of other studies, which found the largest proportions of Ni in the residual phase of urban vegetable garden soils [55], urban soils [52] and urban street dusts [51]. Similarly to Cr, the amounts of Ni found in the reducible phase were generally larger where pseudo-total concentrations were highest, and similarly to Cu, some of the dumpsite soils contained more exchangeable Ni than soils from other locations. 

The reducible fraction was most important for Pb at all sites (Figure 3b), likely reflecting the element’s ability to form stable complexes with Fe-Mn oxides [50,61]. Umoren et al. [54] also found the largest percentage of extractable Pb (75%) associated with the reducible fraction in their study of refuse dump soils in Uyo, southern Nigeria. Similar observations have been reported in other urban areas [52,53]. However, Adeyi et al. [30] found lead fractionation varied between residential soils from different parts of Lagos. In high-income areas, Pb concentration was low (6–17 mg/kg), and the Fe-Mn oxide-bound fraction was dominant; in low-income areas, Pb concentration was higher (90 mg/kg), with higher proportions of Pb found in both more refractory and more labile phases. Particularly large amounts of Pb in the current study were associated with the reducible phase in dumpsite soils A16 (2460 mg/kg) and A19 (688 mg/kg), which is of concern because of the health risk this might pose if the element were mobilized under reducing conditions. Lead associated with the reducible phase can also be liberated by erosion processes of top soils and transported to a new environment, such as road surfaces [62]. 

Zinc was mainly associated with the exchangeable phase, followed by the reducible phase (Figure 3c). A previous study on sediment from three urban rivers and Lagos Lagoon [31] also found that a high proportion (40 to 87%) of Zn content was released in step 1 of the BCR sequential extraction. This is relevant to the current work because trans-urban water bodies are likely to contain considerable amounts of soil-derived material; therefore, trace elements may have similar speciation. A fractionation pattern similar to that reported in the current study were reported for soils and dusts collected in other urban areas [51,52,54,60]. Like Cu, the majority of Zn was present in non-residual forms, which is of concern from the point of view of potential mobilization and transport.

The relative availability of the analytes based on the average proportion found in the exchangeable phase was Fe (1.1%) < Cr (1.3%) < Ni (9.2%) < Pb (12%) < Cu (15%) < Mn (23%) < Zn (48%). Based on the proportion found in the three most labile phases (step 1 + step 2 + step 3), the relative availability was Fe (16%) < Cr (30%) < Ni (46%) < Mn (63%) < Cu (78%) < Zn (80%) < Pb (84%). Elements found to be mainly of lithogenic origin in previous urban soil studies (Fe [50,52] and Cr [4,51]) were similarly less available for mobilization in the Lagos samples; therefore, it might be expected that they would have lesser environmental or human health impact than elements likely of mainly anthropogenic origin (Cu, Pb, Zn). Where concentrations of the urban metals were highest—site A16 for Cu and Pb and site A19 for Zn—they were also more labile, which is clearly of concern.

### 3.3. Human Health Impact

**Figure 4:**
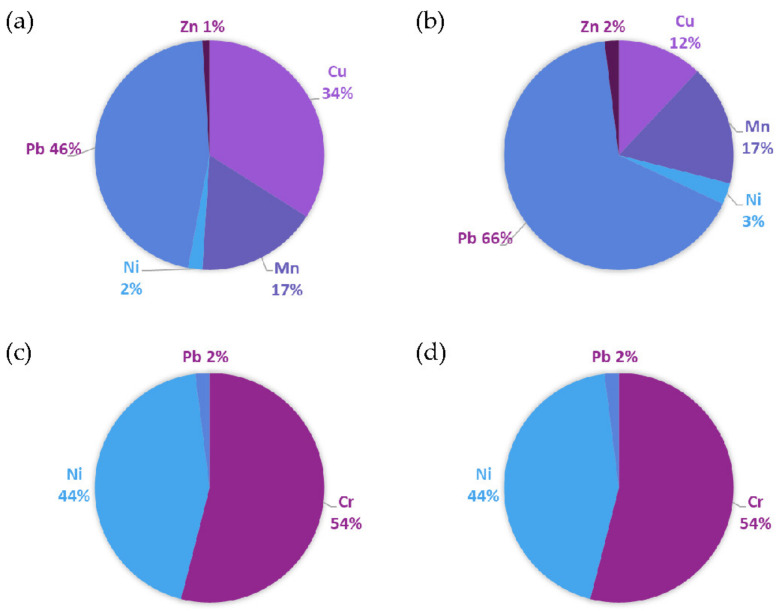
Contribution of PTE to risk: (**a**) non-carcinogenic risks in children, (**b**) non-carcinogenic risks in adults, (**c**) carcinogenic risks in children and (**d**) carcinogenic risks in adults.

Results obtained for human health risk assessment are presented in Table 5 and Figure 4. For non-carcinogenic risk, comparison of HQ values indicates that ingestion was the most significant exposure route for both children and adults, followed by dermal contact and inhalation. The HI (summation of HQ across the three exposure pathways) values were less than 1 for Cr, Ni and Zn, indicating that there is no significant non-carcinogenic risk associated with exposure to the average concentrations of the above PTEs in Lagos urban soils. In contrast, the HI for Cu, Mn and Pb were greater than 1 in children, which suggests that there is a risk of non-carcinogenic health effects. Among the PTEs studied, Pb was the largest contributor to non-carcinogenic risks. This is of particular concern, given the impact that this element can have on children’s development, even at low concentrations. Risk from the other metals followed the order Cu > Mn > Ni > Zn > Cr for children and Mn > Cu > Ni > Zn > Cr for adults (Figure 3). Although similar trends were observed in both groups, non-carcinogenic risk was greater in children than in adults. This is expected, given their lower body mass and immature physiology, as has been reported in many previous studies [63,64].

Carcinogenic risk was calculated for Cr, Ni and Pb based on their respective cancer slope factors (the other analytes are not considered a cancer risk, so slope factor data are not available). For Cr, a slope factor of 0.5 (the lower of the values commonly cited in literature [41]) was used because results of the sequential extraction suggested that the element was not readily available. Risks greater than 1 × 10^−4^ are considered unacceptable, those between 10^−4^ and 10^−6^ acceptable and those less than 1 × 10^−6^ unlikely to lead to any detrimental health outcomes. In the current study, total carcinogenic risk levels for children were greater than those for adults, and values for both Cr and Ni exceeded the threshold for unacceptable risk in both age groups. In contrast, values were in the range of 10^−4^ to 10^−6^ for Pb; therefore, the additional probability of developing cancer over a 70-year lifespan due to exposure to this element at the average concentration found in Lagos soils is considered acceptable.

However, it is important to emphasize that these risk calculations are based on average PTE concentrations. Given the remarkably wide range of analyte concentrations found and the fact that a few values markedly exceeded the mean for each element, it is probable that results overestimate risk for the majority of Lagos residents. 

## 4. Conclusions

Concentrations of Cr, Cu, Fe, Mn, Ni, Pb and Zn varied markedly in soils obtained from public-access areas across the megacity of Lagos. The highest values were found in proximity to known pollution sources, such as dumpsites, but there is evidence that general ambient PTE levels are increasing as rapid urbanization and industrialization occurs. The urban metals—Cu, Pb and Zn—were generally found in more labile forms than elements such as Cr, Fe and Ni and are therefore more susceptible to (re)mobilization and transport under changing environmental conditions. Calculations indicated the presence of non-carcinogenic risk for children, as well as carcinogenic risk for both children and adults, although this is likely associated mainly with sites where PTE concentrations were highest. Nevertheless, further monitoring and assessment of the status of Lagos urban soils is recommended, together with the development and implementation of an appropriate regulatory framework to protect soil quality and public health. 

## Figures and Tables

**Table 1:** BCR sequential extraction [21].

Step	Label	Reagents	Nominal Target Phase(s)
1	Exchangeable	0.11 mol L^−1^ CH_3_COOH	Exchangeable, water- and acid-soluble PTE
2	Reducible	0.5 mol L^−1^ NH_2_OH.HCl at pH 1.5	PTE bound to iron and manganese oxyhydroxides
3	Oxidisable	H_2_O_2_ (85 °C) then 1.0 mol L^−1^ CH_3_COONH_4_ at pH 2	PTE bound to organic matter and sulphides
4	Residual	aqua regia	Remaining PTE not bound within refractory/primary silicates

**Table 2:** Analysis of certified reference materials BCR 143R and BCR 701 (mg/kg dry weight).

Step	Parameter	Cr	Cu	Fe	Mn	Ni	Pb	Zn
BCR 143R								
	Found	422 ± 27	137 ± 3	29,700 ± 12	863 ± 7	297 ± 4	178 ± 3	1110 ± 10
	Certified ^†^	426 ± 12	131 ± 2		858 ± 11	296 ± 4	174 ± 5	1063 ± 16
	Recovery (%)	99	105		101	100	102	104
BCR 701								
Step 1	Found	2.0 ± 1	42 ± 9	66 ± 8	186 ± 25	14 ± 9	3 ± 1	187± 25
Exchangeable	Certified	2.26 ± 0.16	49.3 ± 1.7			15.4 ± 0.9	3.18 ± 0.21	205 ± 6
	Recovery (%)	88	85			91	94	91
Step 2	Found	45 ± 4	124 ± 15	370 ± 3	5 ± 1	25 ± 1	125 ± 8	104 ± 4
Reducible	Certified	45.7 ± 2.0	124 ± 3			26.6 ± 1.3	126 ± 3	114 ± 5
	Recovery (%)	98	100			94	99	91
Step 3	Found	109 ± 9	39 ± 7	246 ± 43	21 ± 0.1	14 ± 0.2	3 ± 0.3	33 ± 1
Oxidisable	Certified	143 ± 7	55 ± 4			15.3 ± 0.9	9.3 ± 20	46 ± 4
	Recovery (%)	76	71			89	32	71
Step 4	Found	80 ± 0.3	44 ± 3	21,400 ± 81	261 ± 20	40 ± 1	13 ± 2	118 ± 9
Residual	Indicative	63 ± 8	39 ± 12			41 ± 4	11 ± 6	95 ± 13
	Recovery (%)	128	114			96	122	124
Σ(steps 1–4)	Found	236	249	22,000	474	93	144	442
	Indicative	253	267			98.7	149	461
	Recovery (%)	93	93			94	97	96

^†^ CRM 143R certified vales are for aqua regia-soluble PTE content, except for Cu, for which only the total content is available.

**Table 3:** Parameters used in risk calculations.

Parameter	Unit	Child	Adult
Ingestion rate (*IR*)	mg/day	200	100
Exposure frequency (*EF*)	days/year	350	350
Exposure duration (*ED*)	years	6	24
Body weight (*BW*)	kg	15	70
Average time (*AT*)	days		
Inhalation rate (*Inh*)	m^3^/day	10	20
Particulate emission factor (*PEF*)	m^3^/kg	1.3 × 10^9^	1.3 × 10^9^
Skin surface area (*SA*)	cm^2^	2100	5800
Soil–skin adherence factor (*AF*)	mg/cm^2^	0.2	0.07
Dermal absorption factor (*ABS*)	none	1	1
For carcinogens		365 × 70	365 × 70
For non-carcinogens		365 × *ED*	365 × *ED*

**Table 4:** Pseudo-total PTE concentrations in Lagos urban soils (mg/kg dry weight).

Sampling Site	Land Use	pH	% LOI	Cr	Cu	Fe	Mn	Ni	Pb	Zn
A1	PO	7.2	3.6	22 ± 1	25 ± 4	12,600 ± 485	233 ± 22	8 ± 1	87 ± 15	239 ± 36
A2	PO	10	5.0	25 ± 2	27 ± 8	12,000 ± 1310	179 ± 26	6 ± 0.1	26 ± 5	175 ± 4
A3	PO	8.1	1.4	30 ± 2	10 ± 2	10,000 ± 178	212 ± 59	8 ± 4	24 ± 4	99 ± 45
A4	PO	7.0	0.36	*19 ± 1*	*8.0* ± *1*	*7460* ± *284*	*135* ± *38*	*4* ± *0.2*	*10* ± *1*	*61* ± *3*
A5	PO	7.0	0.92	34 ± 12	29 ± 12	14,100 ± 2110	199 ± 49	8 ± 18	41 ± 12	165 ± 25
A6	PO	6.5	0.11	51 ± 1	18 ± 1	22,300 ± 480	359 ± 19	16 ± 0.2	57 ± 3	165 ± 3
A7	PO	8.3	2.2	38 ± 6	20 ± 1	11,100 ± 1680	159 ± 17	8 ± 1	29 ± 5	132 ± 17
A8	IE	7.0	1.4	49 ± 4	71 ± 6	28,600 ± 4280	375 ± 32	17 ± 5	400 ± 154	1080 ± 123
A9	IE	8.1	0.17	111 ± 18	71 ± 8	60,200 ± 1060	437 ± 73	39 ± 5	144 ± 14	433 ± 37
A10	IE	5.8	4.3	175 ± 12	759 ± 406	146,000 ± 15,600	2570 ± 311	109 ± 9	536 ± 62	3240 ± 475
A11	RT	5.9	1.6	71 ± 16	218 ± 7	46,300 ± 1780	413 ± 14	29 ± 3	182 ± 10	241 ± 37
A12	RT	7.4	2.0	94 ± 4	168 ± 2	69,100 ± 787	1220 ± 39	44 ± 3	321 ± 14	1200 ± 35
A13	RT	6.9	0.67	46 ± 3	243 ± 17	36,100 ± 8370	401 ± 70	21 ± 2	212 ± 53	1220 ± 283
A14	DS	7.0	2.6	290 ± 42	182 ± 64	47,600 ± 3810	899 ± 34	139 ± 24	76 ± 4	880 ± 202
A15	DS	7.1	0.85	79 ± 32	133 ± 19	41,100 ± 2730	566 ± 59	29 ± 8	153 ± 29	546 ± 6
A16	DS	8.0	1.2	**1830** ± **76**	**11,700** ± **1780**	**166,000** ± **2530**	1540 ± 62	**1050** ± **240**	**4340** ± **974**	2810 ± 70
A17	DS	8.3	0.07	202 ± 30	82 ± 20	99,900 ± 4250	1600 ± 107	38 ± 3	102 ± 26	353 ± 12
A18	DS	8.6	0.77	47 ± 19	108 ± 14	14,700 ± 1020	261 ± 75	12 ± 1	315 ± 45	511 ± 222
A19	DS	8.0	0.16	108 ± 13	611 ± 153	57,600 ± 5230	753 ± 82	50 ± 68	802 ± 305	**5620** ± **362**
A20	DS	8.2	0.08	602 ± 53	108 ± 21	52,600 ± 5340	**6100** ± **1750**	306 ± 31	22 ± 7	520 ± 20
Mean		7.5	1.5	196	755	49,600	930	97	394	985
Dutch target [44]				100	36			35	85	140
Dutch intervention [44]				380	190			210	530	720

PO = parks, gardens and open spaces; IE = industrial estates; RT = railway terminals; DS = dumpsites. The lowest concentration for each metal is indicated in italics, and the highest concentration is indicated in bold.

**Table 5:** Human health risk assessment for children and adults.

Element	HQ_ing_	HQ_inh_	HQ_dermal_	HI Children	HQ_ing_	HQ_inh_	HQ_dermal_	HI Adult	CR_total_ Children	CR_total_ Adult
Cr	1.67 × 10^−2^	3.23 × 10^−5^	3.51 × 10^−3^	5.21 × 10^−3^	1.79 × 10^−4^	1.23 × 10^−5^	6.49 × 10^−4^	8.40 × 10^−4^	**3.33 × 10^−4^**	**2.33 × 10^−4^**
Cu	2.60 × 10^0^	2.96 × 10^−5^	5.47 × 10^−1^	**3.15 × 10^0^**	2.79 × 10^−2^	1.32 × 10^−5^	1.18 × 10^−1^	1.46 × 10^−1^		
Mn	4.95 × 10^−1^	3.05 × 10^−6^	1.04 × 10^0^	**1.53 × 10^0^**	5.30 × 10^−2^	9.26 × 10^−7^	1.52 × 10^−1^	2.05 × 10^−1^		
Ni	6.19 × 10^−2^	7.70 × 10^−6^	1.30 × 10^−1^	1.92 × 10^−1^	6.63 × 10^−3^	2.92 × 10^−6^	2.38 × 10^−2^	3.05 × 10^−2^	**2.76 × 10^−4^**	**1.93 × 10^−4^**
Pb	1.39 × 10^0^	3.56 × 10^−6^	2.93 × 10^0^	**4.33 × 10^0^**	1.49 × 10^−1^	1.60 × 10^−6^	6.38 × 10^−1^	7.88 × 10^−1^	1.13 × 10^−5^	7.96 × 10^−6^
Zn	4.19 × 10^−2^	1.28 × 10^−5^	8.81 × 10^−2^	1.30 × 10^−1^	4.49 × 10^−3^	5.64 × 10^−6^	1.87 × 10^−2^	2.32 × 10^−2^		

Values in bold indicate an HI value > 1 or a CR value > 1 × 10^−4^.

## Data Availability

Data are available on request from abimbola.famuyiwa@gmail.com.

## Supplementary Materials

The following supporting information can be downloaded at: https://www.mdpi.com/article/10.3390/toxics10040154/s1, Table S1: Results for sequential extraction of chromium (mg/kg); Table S2: Results for sequential extraction of copper (mg/kg); Table S3: Results for sequential extraction of iron (mg/kg); Table S4: Results for sequential extraction of manganese (mg/kg); Table S5: Results for sequential extraction of nickel (mg/kg); Table S6: Results for sequential extraction of lead (mg/kg); Table S7: Results for sequential extraction of zinc (mg/kg).
