# Supplementary material for: Potentially Toxic Elements in Urban Soils from Public-Access Areas in the Rapidly Growing Megacity of Lagos, Nigeria
Source: Toxics. 2022 Mar 23;10(4):154. doi: 10.3390/toxics10040154 (PMC9025973; doi:10.3390/toxics10040154)
Supplement: Supplementary file 1 [file toxics-10-00154-s001.zip › toxics-1600747-supplementary.pdf]

toxics MDPI

Supplementary Materials: Potentially Toxic Elements in Urban Soils from Public-Access Areas in the Rapidly Growing Megacity of Lagos, Nigeria

Abimbola O. Famuyiwa, Christine M. Davidson, Sesugh Ande and Aderonke O. Oyeyiola

Table S1. Results for sequential extraction of chromium (mg/kg).

	Sequential extraction steps					Pseudototal	Recovery†
	**Residual**	**Oxidisable**	**Reducible**	**Exchangeable**	**Sum**	**(PT)**	**%**
A1	10.5	3.28	3.51	1.02	18.3	22.4	81.8
A2	13.5	3.25	1.81	0.965	19.5	24.7	79.2
A3	23.2	2.99	1.39	0.333	27.9	30.4	91.6
A4	12.2	1.69	0.66	0.282	14.8	18.8	78.5
A5	21.4	2.86	1.04	0.367	25.7	34.3	75
A6	35.3	5.91	1.33	0.0852	42.6	51	83.5
A7	26.5	3.89	1.37	0.479	32.3	37.9	85.1
A8	42	3.11	4.83	0.447	50.4	49.2	102
A9	66.8	8.42	5.66	0.626	81.5	111	73.2
A10	116	13.9	31.7	1.27	162	175	93
A11	34	11.8	4.48	0.0403	50.4	71	71
A12	42.6	27.7	13.5	1	84.9	93.6	90.7
A13	33.3	7.16	5.08	0.145	45.7	46.2	99
A14	170	7.97	76.6	1.35	256	290	88.2
A15	35	5.42	10.7	0.577	51.6	78.7	65.6
A16	1180	235	1020	37.3	2470	1830	135
A17	80	9.15	109	1.09	199	202	98.3
A18	22	4.54	7.58	0.469	34.6	47.5	72.9
A19	52.2	23.7	9.22	0.205	85.3	108	79
A20	430	31.3	95.7	5	562	602	93.3

† Recovery = S(steps 1–4)/PT × 100.

Table S2. Results for sequential extraction of copper (mg/kg).

	Sequential Extraction Eteps					Pseudototal	Recovery†
	**Residual**	**Oxidisable**	**Reducible**	**Exchangeable**	**Sum**	**(PT)**	**%**
A1	4.76	3.67	10.3	2.18	20.9	25.2	83.1
A2	3	15	5.56	1.91	25.5	27	94.3
A3	3.27	1	3	1.71	11.1	10.4	107
A4	1.25	0.961	2.09	2.14	6.44	8.36	77.1
A5	4.69	6.02	6.93	3.9	18.5	28.5	64.9
A6	10.1	3.94	5.72	0.429	20.2	18	112
A7	3.04	3.94	4	3.07	14	20.3	70
A8	10.1	4	18.6	9.96	42.7	71.1	60
A9	11.7	7.16	18.3	9.63	46.7	70.5	66.2
A10	102	22	363	102	589	759	77.5
A11	35.5	75	54.8	0.768	166	218	76
A12	37.5	51.6	58.8	4	152	168	90.5
A13	30	56	156	16.6	259	243	106
A14	24	4	70	52.6	151	182	82.7
A15	15.2	6	81.2	32.6	135	133	101
A16	2150	579	11,300	3230	16,700	11,700	142
A17	21.4	2.93	49	8.69	82.1	82.4	99.6
A18	8	13	74	24	119	108	110
A19	126	199	257	10	593	611	97
A20	27.9	8.11	38.2	10.9	85.1	108	78.9

† Recovery = S(steps 1–4)/PT × 100.

Table S3. Results for sequential extraction of iron (mg/kg).

	Sequential extraction steps					Pseudototal	Recovery†
	**Residual**	**Oxidisable**	**Reducible**	**Exchangeable**	**Sum**	**(PT)**	**%**
A1	9800	98.2	2330	106	12,300	12,600	97.5
A2	7230	49.1	1130	223	8450	12,000	70.4
A3	7860	36.3	748	5.93	8650	10,000	86.2
A4	5480	22	463	39	6010	7460	80.6
A5	12,200	41	683	231	13,100	14,100	93.1
A6	17,500	71.5	949	2.93	18,600	22,300	83.4
A7	8760	41.1	740	285	9820	11,100	88.4
A8	20,200	28	2500	141	22,900	28,600	79.9
A9	33,700	79.1	2280	193	36,200	60,200	60.2
A10	125,000	196	19,300	113	144,000	146,000	98.8
A11	29,000	914	3430	1.05	33,300	46,300	72
A12	51,900	904	8170	97	61,100	69,100	88.4
A13	25,000	286	3240	6.54	28,600	36,100	79.2
A14	39,400	99.8	8030	298	47,900	47,600	101
A15	30,500	83.2	4840	158	35,600	41,100	86.6
A16	119,000	697	44,800	872	166,000	166,000	99.7
A17	58,900	139	38,600	2610	100,000	99,900	100
A18	9190	75.3	3700	288	12,600	14,700	85.8
A19	40,000	145	4810	1.8	44,900	57,600	78
A20	38,000	145	11,000	2360	51,500	52,600	97.9

† Recovery = S(steps 1–4)/PT × 100.

Table S4. Results for sequential extraction of manganese (mg/kg).

	Sequential Extraction Steps					Pseudototal	Recovery†
	**Residual**	**Oxidisable**	**Reducible**	**Exchangeable**	**Sum**	**(PT)**	**%**
A1	56.2	7.1	101	65.6	230	233	99
A2	48.3	4.48	43	40.2	129	179	72.3
A3	39.6	3.28	58.8	39.7	141	212	66.8
A4	35	2.72	20	28.2	89	135	65.9
A5	68.2	6	31	43.1	157	199	78.8
A6	93.3	6	114	107	320	359	89.1
A7	70.2	6	41.1	42	161	159	101
A8	152	10	166	76.7	440	375	117
A9	146	10.6	153	56.1	365	437	83.6
A10	1180	72	963	393	2630	2570	102
A11	126	14.5	143	37	330	413	79.9
A12	232	17.3	687	169	1110	1220	90.8
A13	126	9.64	107	95.6	338	401	84.5
A14	345	16.3	344	269	974	899	108
A15	198	32.1	183	118	531	566	93.8
A16	690	91.1	384	395	1560	1530	102
A17	588	46.3	550	382	1570	1600	98
A18	42.4	5	132	58.6	238	261	91.3
A19	141	11.8	381	165	698	753	92.7
A20	3280	140	1220	562	5710	6100	93.7

† Recovery = S(steps 1–4)/PT × 100.

Table S5. Results for sequential extraction of nickel (mg/kg).

	Sequential Extraction Steps					Pseudototal	Recovery†
	**Residual**	**Oxidisable**	**Reducible**	**Exchangeable**	**Sum**	**(PT)**	**%**
A1	3.33	1.07	1.65	1.44	7.49	7.72	97.1
A2	3.29	1.46	0.63	0.8	6.18	6.14	101
A3	3.15	0.909	1.18	0.429	5.67	8.19	69.1
A4	2.39	0.803	0.694	0.3	4.19	4.49	93.3
A5	4.44	1.07	0.882	0.712	7.1	8	88.8
A6	10.2	1.35	1.3	0.584	13.4	16	83.9
A7	4.21	1.06	0.492	0.997	6.76	7.57	89.3
A8	7	1.27	3.73	1.3	13.3	16.6	80.3
A9	15.9	2.2	7	2	27.1	38.7	69.9
A10	49.9	5.8	47.2	14.4	117	109	108
A11	15.2	5.27	3.66	0.573	24.7	29.4	83.9
A12	27.6	5.05	8.03	4	44.7	44.4	101
A13	11	2.01	5.38	2.63	21	21	99.9
A14	90.1	2.01	36.9	3.1	132	139	95.2
A15	11.9	1.73	7.74	1.99	23.3	29	80.5
A16	321	38.5	614	106	1080	1050	103
A17	17.2	2.22	23	2	50.5	38	133
A18	6	1.3	3.87	2	13.2	12	110
A19	24	4.02	16	2.92	45	49.5	91
A20	70.5	4.11	35	8.95	119	306	38.8

† Recovery = S(steps 1–4)/PT × 100.

Table S6. Results for sequential extraction of lead (mg/kg).

	Sequential Extraction Steps					Pseudototal	Recovery†
	**Residual**	**Oxidisable**	**Reducible**	**Exchangeable**	**Sum**	**(PT)**	**%**
A1	5.74	2.55	32.4	1.49	42.2	87.3	48.4
A2	6.87	3.66	14	2.78	25	26.4	94.8
A3	4.22	2	17.3	2.7	25.6	24.3	105
A4	2.08	0.534	5.34	2.39	10.3	10.4	99.1
A5	4.94	2	17.3	5.63	29.3	40.8	71.9
A6	13.7	3.03	30.1	1.29	48.1	57	84.3
A7	4	1.05	16.3	9	29	28.7	101
A8	13	102	101	99	315	400	78.5
A9	36.8	12.9	64.3	10.7	125	144	86.5
A10	47	244	367	113	771	536	144
A11	56.9	33.8	216	1.78	308	182	169
A12	60.2	18.5	211	2	292	321	90.9
A13	23.4	9	139	1.91	175	212	82.4
A14	10.5	2.23	39.8	6.32	58.9	76.5	77
A15	18	5.25	89.1	14	124	153	81.3
A16	113	39	2460	157	2770	4340	63.8
A17	11	1.97	46	5.91	64.9	102	63.8
A18	7.63	3.5	184	102	284	315	90.2
A19	104	66.1	688	39	984	802	123
A20	4.53	0.608	10.3	0.488	15.9	21.6	73.9

† Recovery = S(steps 1–4)/PT × 100.

Table S7. Results for sequential extraction of zinc (mg/kg).

	Sequential Extraction Steps					Pseudototal	Recovery†
	**Residual**	**Oxidisable**	**Reducible**	**Exchangeable**	**Sum**	**(PT)**	**%**
A1	30.7	12.1	53.2	134	230	239	96.4
A2	29.8	23.2	20.6	97.7	171	175	98
A3	32	5.73	14.8	27.4	79.9	98.7	81
A4	17	4	6.19	29.8	57	61	93.6
A5	23.5	10.8	14.3	88.7	137	165	83.3
A6	41.1	15.1	30.2	59	145	165	88.1
A7	25.2	11.4	15.4	76.8	129	132	97.4
A8	98	30.8	256	600	1030	1090	95
A9	62.5	26.1	70.6	177	336	433	77.5
A10	633	129	842	1130	2730	3250	84.3
A11	71.6	28.8	41.3	20.1	162	241	67.1
A12	139	79.7	346	726	1290	1200	108
A13	150	37.1	287	452	926	1220	75.7
A14	80	21.5	154	368	624	880	70.9
A15	86.9	25.1	171	220	502	546	92
A16	218	81.1	1010	1080	2400	2810	85.4
A17	67.3	19.1	108	130	325	353	91.9
A18	34.2	12.9	147	218	412	511	80.7
A19	712	230	1470	2330	4740	5620	84.3
A20	108	103	188	174	572	520	110

† Recovery = S(steps 1–4)/PT × 100.
